# College and Hospital Notes

**Published:** 1896-09

**Authors:** 


### College and Hospital Notes.

Dr. I. N. Love, for many years a highly honored and popular teacher in Marion-Sims college of medicine, of St. Louis, having seen fit, under pressure of professional and editorial duties, to resign his chair, the faculty has published the following minutes :

WHEREAS, Dr. I. N. Love has found it incumbent upon him to sever his connection with the Marion-Sims college of medicine, the members of the faculty of that institution embrace this occasion to express their appreciation of his past services, and to extend to him their hope that in all his future connections he will find both pleasure and profit.

B. M. HYPES,
R. C. ATKINSON,
C. BARCK, *Committee.*

---

THE Kentucky School of Medicine, at a recent meeting of its faculty, announced the following appointments: Dr. Louis Frank, lecturer on clinical and operative gynecology; Dr. Henry E. Tuley, lecturer on obstetrics; Dr. T. C. Evans, lecturer on ophthalmology, otology and laryngology; Dr. Carl Weidner, lecturer on physiology; Dr. W. Ed. Grant, lecturer on anatomy; Dr. Ewing Marshall, lecturer on physical diagnosis.

